# Predictors of gastrointestinal complaints in patients on metformin therapy

**DOI:** 10.1515/med-2023-0871

**Published:** 2023-11-29

**Authors:** Branislava Raičević, Slobodan Janković

**Affiliations:** Clinical Pharmacology, Faculty of Medical Science, University of Kragujevac, Kragujevac, Serbia; Pharmacology and Toxicology Department, Faculty of Medical Sciences, University of Kragujevac, Kragujevac, Serbia

**Keywords:** metformin intolerance, risk factors, statins, anaemia

## Abstract

Although being very effective in the treatment of diabetes and a few other conditions, metformin (MTF) cannot be tolerated by many patients due to gastrointestinal (GI) complaints. A number of risk factors for intolerance were identified, but many are still controversial or uninvestigated. The aim of this study was to further investigate possible risk factors for the occurrence of GI complaints in patients on MTF therapy. A cross-sectional design was used for this multicentric study on adult patients visiting 50 community pharmacies in Montenegro. The patients were surveyed by semi-structured questionnaire after a service of a pharmacist was delivered, and their drugs dispensed. Uni- and multi-variate regression methods were used for processing the data.

In total 330 patients participated in the study. A higher body mass index (OR = 1.113, *p* = 0.003), living at a higher altitude (OR = 1.725, *p* = 0.000), anaemia (OR = 4.221, *p* = 0.008), and intestinal infection in the last 3 months (OR = 2.801, *p* = 0.006) increased the risk of GI complaints in patients on MTF therapy, while the use of statins was protective (OR = 0.204, *p* = 0.016). Each case of MTF intolerance should be carefully investigated for risk and protective factors, which could be potentially eliminated or augmented, respectively, and MTF withdrawal avoided.

## 1 Introduction

### 1.1 Definitions and epidemiology

Metformin (MTF) is an oral antidiabetic drug that, due to its effectiveness and safety, as well as its relatively low price, represents the first pharmacological therapeutic line in the treatment of type 2 diabetes according to the guidelines of the European and American diabetes associations [1,2]. In recent years, MTF has been the subject of numerous studies that indicate the beneficial effect of MTF in many other diseases, such as numerous types of cancer, obesity, and cardiovascular and neurodegenerative diseases, as well as liver and kidney diseases [2]. However, MTF treatment is often (20–30%) associated with gastrointestinal (GI) adverse effects (AEs) [3,4]. This GI intolerance negatively affects quality of life and compliance, and 5% of patients discontinue therapy [4,5]. GI problems mostly occur at the beginning of therapy, but there are studies that indicate the occurrence of GI complaints and after a long time of drug use [5].

### 1.2 Known risk factors

The mechanism underlying MTF-induced GI intolerance is still unclear. There are several hypotheses trying to give an explanation: stimulation of intestinal serotonin secretion, changes in incretins and glucose metabolism, and malabsorption of bile salts [4,5]. Not much is known about risk factors for the occurence of GI intolerance, too. There are just a few studies that show the possible association of certain factors and the occurrence of GI AEs of MTF [6]. An observational study comparing 83 patients who discontinued MTF therapy due to GI AEs with 332 age- and sex-matched controls indicated a possible association between GI intolerance to MTF and rate of ischemic heart disease, left-handedness, ABO blood groups, and iron load [7]. Another study found an association between GI AEs of MTF and characteristics of large bowel microbiota [8]. There are also claims that females are more often intolerant to MTF, but more evidence is needed for this to be confirmed [9]. It is of vital importance to reveal and then control the factors associated with GI AEs of MTF, or otherwise a number of patients will stop taking MTF due to intolerance, depriving themselves of very effective and convenient drug.

The aim of this study was to further investigate possible risk factors for the occurrence of GI complaints in patients on MTF therapy.

## 2 Methods

### 2.1 The study design and population

**Figure 1:**
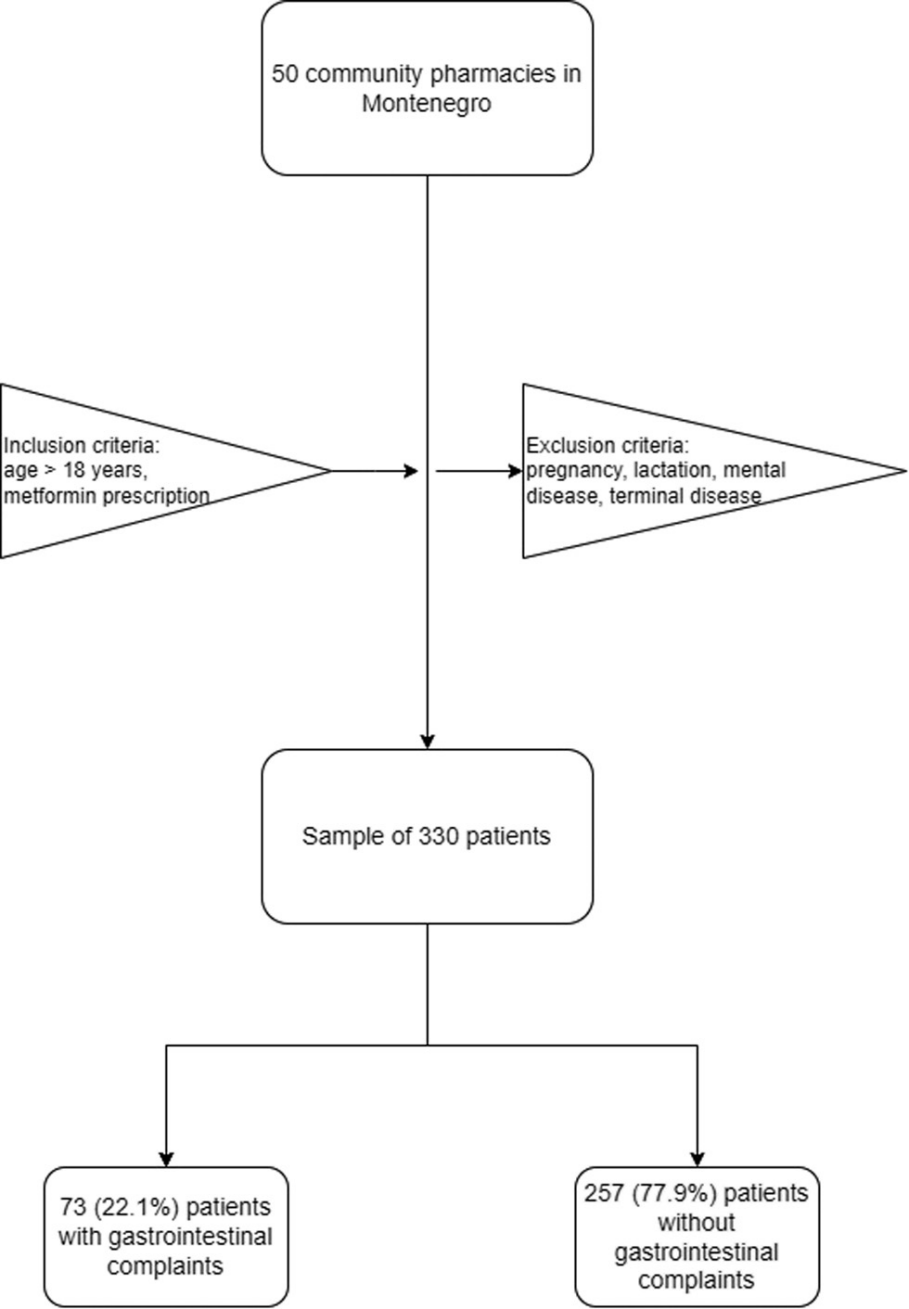
The study flowchart.


The research was conducted as a cross-sectional study on adult patients in 50 community pharmacies out of a total of 250 pharmacies in the territory of the whole of Montenegro (Figure 1). The study was conducted from June 2022 until October 2022 on a convenient sample of patients who visited pharmacies where the researchers worked on the dispensing of medicines. Inclusion criteria were: age over 18 years, possession of MTF prescription, permanent residence in Montenegro, and signed patient consent form. Excluded from the study were pregnancy and lactation, patients prescribed with medication for psychiatric illnesses or dementia, patients with chronic disease in the terminal phase, as well as patients who came to the pharmacy for medication due to acute conditions.

**Ethical approval:** Before its start, the study was approved by the Ethics Committee of the Faculty of Medicine, University of Montenegro, and the Ethics Committee of the Pharmaceutical Chamber of Montenegro. The patients in the study were treated according to the principles of Helsinki Declaration on the protection of human subjects of clinical investigations and to those of Good Clinical Practice.

### 2.2 The study procedures

The study data were collected from patients in the pharmacies they visited using questionnaires filled out by researchers based on patients’ verbal responses. Before completing the questionnaire, patients were offered brief information about the key elements of their participation, and then patients would be included if they signed a consent to participate. The survey collected patient demographic data, data on GI complaints related to taking MTF, comorbidities, and data on concomitant therapy and habits.

### 2.3 The sample size

The minimum sample size of 194 patients required to achieve a study power of at least 80%, with a statistical error of the first type (alpha) of 0.05, was calculated based on the *z*-test (difference between two independent proportions) and the expected difference in predictor frequency between the group with and the group without GI complaints of 20%.

### 2.4 Statistics

After testing the normality of the distribution of values of continuous variables by Kolmogorov–Smirnov test, those with normal distribution were described by mean and standard deviation, and those without were described by median and interquartile range. The study groups were compared in terms of continuous variables by Student’s *T*-test for independent samples if normally distributed and by Mann–Whitney *U* test if not following a normal distribution. Categorical variables were described by rates and percentages, and differences between the study groups were tested by the Chi-square test, or by the Fisher exact test if the frequency of one of the the categories was below 5.

To explore the influence of independent or confounding variables adjusted for other predictors on the binary categorical outcome, multivariate binary logistic regression was used, after ensuring previously that its assumptions were met: linearity, absence of outliers, independence of variables, and absence of collinearity. The final model was obtained by backward deletion procedure. Quality of the final model was examined with the Hosmer–Lemeshow test. The extent to which the final binary logistic regression model explained the outcome was estimated by Nagelkerke’s pseudo *R* ^2^ and Cox and Snellen’s pseudo *R* ^2^. The statistical tests were considered significant if the probability of the null hypothesis was below 0.05.

## 3 Results

### 3.1 Descriptive statistics

The questionnaire response rate was 89%. In total, 330 patients completed the study, of whom 73 (22.1%) had GI complaints that accompanied the use of MTF, and 257 (77.9%) of them did not have GI complaints after the introduction of MTF into therapy. The types of GI complaints that accompanied the use of MTF were distributed as follows: nausea occurred in 11 (15.1%) patients, diarrhea in 17 (23.3%) patients, nausea with abdominal pain in 6 (8.2%), flatulence in 9 (12.3%) patients, nausea, abdominal pain and diarrhea in 25 (34.3%) patients, and abdominal pain combined with flatulence in 5 (6.8%) individuals. In the largest number of patients, 42 of them (57.5%), the complaints passed spontaneously, and the patients continued taking MTF; 26 (35.6%) patients required the use of drugs to suppress GI complaints but still continued to take the drug. Only 5 patients (6.9%) were forced to discontinue MTF due to intolerance to GI complaints; after discontinuation of the drug, GI complaints disappeared in all patients. GI complaints appeared on average after 6.3 weeks from the start of taking MTF and lasted on average 33.4 weeks. On a scale from 1 to 10, the average intensity of GI complaints associated with the use of MTF in our sample was 4.3 ± 2.2 points. Detailed characteristics of the groups of patients with and without GI complaints are shown in Table 1.

**Table 1:** Characteristics of patients by study groups

Study variable	Patients with GI complaints (*n* = 73)	Patients without GI complaints (*n* = 257)	Null hypothesis probability*
Age (years)	62 (19.5)	65 (13.0)	0.033^§^
Gender (male/female)	24/49 (32.9%/67.1%)	117/138 (45.9%/54.1%)	0.054
BMI (kg/m^2^)	27.5 (6.4)	26.4 (5.2)	0.047
MTF daily dose (mg)	1000.0 (1000.0)	1000.0 (1000.0)	0.594
Number of individual doses per day	2.0 (1.0)	2.0 (1.0)	0.384
MTF therapy (months)	60.0 (99.0)	60.0 (96.0)	0.263
Altitude of the patient’s residence (m)	173.0 (629.0)	44.0 (36.0)	0.003^§^
Charlson Comorbidity Index	3.0 (2.0)	3.0 (2.0)	0.193
Systolic blood pressure	130.0 (20.0)	130.0 (15.0)	0.707
Diastolic blood pressure	85.0 (10.0)	80.0 (10.0)	0.715
Physical activity in hours per week	7.0 (10.0)	10.0 (16.0)	0.013^§^
Marital status: married/not married	59 (80.8%)/14 (19.2%)	216 (84.0%)/41 (16.0%)	0.514
Zanimanje: not actively working/office jobs/manual labor jobs	40 (54.8%)/12 (16.4%)/21 (28.8%)	150 (58.4%)/24 (9.3%)/83 (32.3%)	0.226
Education: elementary/high school/higher education	4 (5.4%)/40 (54.8%)/29 (39.8%)	17 (6.6%)/150 (58.7%)/89 (34.7%)	0.313
Residency: town/village	70 (95.9%)/3 (4.1%)	246 (94.1%)/10 (3.9%)	1.000
Residency: continental/seaside	54 (74.0%)/19 (26.0%)	148 (57.8%)/108 (42.2%)	0.012^§^
Immediate/delayed release MTF	54 (74.0%)/19 (26.0%)	193 (75.1%)/64 (24.9%)	0.845
MTF monotherapy/fixed combination	50 (68.5%)/23 (31.5%)	207 (80.5%)/50 (19.5%)	0.052
Indication for MTF: diabetes type 2/other	62 (84.9%)/11 (15.1%)	215 (83.7%)/42 (16.3%)	0.794
Missed doses per week: none/1–2/≥3	51 (69.9%)/16 (21.9%)/6 (8.2%)	202 (78.6%)/46 (17.9%)/9 (3.5%)	0.147
Using food supplements in the last 3 months: yes/no	24 (32.9%)/49 (67.1%)	77 (30.0%)/180 (70.0%)	0.633
Using statins: yes/no	3 (4.1%)/70 (95.9%)	42 (16.3%)/215 (83.7%)	0.006^§^
Using ACE inhibitors: yes/no	33 (45.2%)/40 (54.8%)	101 (39.3%)/156 (60.7%)	0.365
Prior surgery: yes/no	25 (34.2%)/48 (65.8%)	80 (31.1%)/177 (68.9%)	0.614
Prior injuries: yes/no	8 (11.0%)/65 (89.0%)	38 (14.8%)/219 (85.2%)	0.405
Any drug allergy: yes/no	9 (12.3%)/64 (87.7%)	38 (14.8%)/219 (85.2%)	0.596
Any allergy: yes/no	12 (16.4%)/61 (83.6%)	25 (9.7%)/232 (90.3%)	0.109
Intestinal infections in last 3 months: yes/no	20 (27.4%)/53 (72.6%)	28 (10.9%)/229 (89.1%)	0.000^§^
Inflammatory bowel disease: yes/no	2 (2.7%)/71 (97.3%)	0 (0.0%)/257 (100.0%)	0.048^§^
Migraine or cluster headache in the last 3 months: yes/no	8 (11.0%)/65 (89.0%)	20 (7.8%)/237 (92.2%)	0.390
Tension headache in the last 3 months: yes/no	18 (24.7%)/55 (75.3%)	33 (12.9%)/224 (87.1%)	0.014^§^
GERD: yes/no	26 (35.6%)/47 (64.4%)	46 (17.9%)/211 (82.1%)	0.001^§^
Peptic ulcer: yes/no	2 (2.7%)/71 (97.3%)	4 (1.6%)/253 (98.4%)	0.617
*Helicobacter pylori*: yes/no	4 (5.5%)/69 (94.5%)	5 (1.9%)/252 (98.1%)	0.113
Anemia: yes/no	10 (13.7%)/63 (86.3%)	10 (3.9%)/247 (96.1%)	0.002^§^
Kidney disease: yes/no	7 (9.6%)/66 (90.4%)	13 (5.1%)/244 (94.0%)	0.152
Liver disease: yes/no	4 (5.5%)/69 (94.5%)	14 (5.4%)/243 (94.6%)	1.000
Smoking: no/yes/ex smoker	44 (60.3%)/18 (24.7%)/11 (15.1%)	159 (61.9%)/64 (24.9%)/34 (13.2%)	0.920
Drinking alcohol: no/yes/ex drinker	63 (86.3%)/8 (11.0%)/2 (2.7%)	212 (82.5%)/39 (15.2%)/6 (2.3%)	0.659
Drinking coffee: yes/no	60 (82.2%)/13 (17.8%)	199 (77.4%)/58 (22.6%)	0.382
Vegetarian or vegan: yes/no	1 (1.4%)/72 (98.6%)	4 (1.6%)/253 (98.4%)	1.000
Having special diet in the last 3 months: yes/no	11 (15.1%)/62 (84.9%)	20 (7.8%)/237 (92.2%)	0.060
Religious fasting in the last 3 months: yes/no	9 (12.3%)/64 (87.7%)	24 (9.3%)/233 (90.7%)	0.452
Dominant source of proteins: fish/red meat/both fish and red meat/neither fish nor red meat	9 (12.3%)/35 (47.9%)/23 (31.5%)/6 (8.2%)	39 (15.2%)/97 (37.7%)/102 (39.7%)/19 (7.4%)	0.415
Eating spicy, salty and hot food: yes/no	31 (42.5%)/42 (57.5%)	92 (35.8%)/165 (64.2%)	0.298
Adding salt or spices to already cooked food: yes/no	24 (32.9%)/49 (67.1%)	72 (28.0%)/185 (72.0%)	0.420
Sufficiently chewing food: yes/no	51 (69.9%)/22 (30.1%)	179 (69.6%)/78 (30.4%)	0.972
Eating fruits together with their seeds: yes/no	36 (49.3%)/37 (50.7%)	137 (53.3%)/120 (46.7%)	0.547

*Values of continuous variables were compared with non-parametric ones Mann–Whitney *U* test, because the variables were not normally distributed, while categorical variables were compared with the Chi-square test or Fisher’s test (in case the frequency of a category was less than 5). For continuous variables, variable values are presented using median and interquartile range.

^§^Statistically significant difference.

GERD – Gastroesophageal reflux disease.

### 3.2 Multivariate analysis

Multivariate binary logistic regression was used to investigate the association of independent and confounding variables with GI AEs of MTF. The model was built by backward conditional deletion method, beginning with the following potential predictors: age, sex, body mass index (BMI), daily dose of MTF, number of daily doses, length of MTF therapy, Box-Cox transformed altitude of the patient’s residence ([altitude ^ lambda − 1]/lambda, lambda = −0.15), Charlson Comorbidity Index, blood pressure, physical activity per week, marital status, occupation, education, residency, immediate/delayed release MTF, MTF formulation, diagnosis, number of missed doses per week, using food supplements in the last 3 months, using statins, using angiotensin-converting enzyme (ACE) inhibitors, prior surgery, prior injury, allergy, intestinal infections in last 3 months, inflammatory bowel disease, migraine or cluster headache in last 3 months, tension headache in the last 3 months, GERD, peptic ulcer, *Helicobacter pylori*, anemia, kidney disease, liver disease, smoking, drinking alcohol, drinking coffee, vegetarian or vegan, having special diet in the last 3 months, religious fasting in the last 3 months, dominant source of proteins in food, eating spicy, salty and hot food, adding salt or spices to already cooked food, sufficiently chewing food, and eating fruits together with their seeds. The assumptions of logistic regression were met: binary outcome (GI AEs or not), observations were independent, no multicollinearity (variance inflation factor – VIF was below 1.5 for all predictors), sufficient size of the sample, and no extreme outliers. The linear relationship between explanatory variables and the logit of the outcome was tested and confirmed for all continuous variables by the Box-Tidwell test (*p* > 0.05). The variables included in the final model of binary logistic regression are shown in Table 2; the model was a satisfactory fit of the data: Hosmer and Lemeshow test was 11.632 (df = 8, *p* = 0.168), Cox and Snell *R* square 0.166, and Nagelkerke *R* square 0.254.

**Table 2:** Predictors of GI complaints in patients on MTF therapy

Risk factors	Raw OR (95% CI)	*p*	Adjusted OR (95% CI)	*p*
BMI	1.052 (0.992–1.116)	0.091	1.113 (1.037–1.194)	0.003
Transformed altitude	1.605 (1.224–2.105)	0.001	1.725 (1.276–2.333)	0.001
Using statins	0.219 (0.066–0,730)	0.013	0.204 (0.056–0.747)	0.016
Intestinal infection in the last 3 months	3.086 (1.616–5.894)	0.001	2.801 (1.346–5.829)	0.006
Anemia	3.921 (1.564–9.830)	0.004	4.221 (1.456–12.236)	0.008

CI – confidence interval; OR – odds ratio.

## 4 Discussion

This study showed that a higher BMI, living at a higher altitude, anaemia, and intestinal infection in the last 3 months, increases the risk of GI complaints in patients on MTF therapy, while the use of statins is protective. While anaemia and previous intestinal infection increase the risk by 4.2 and 2.8 times, respectively, each additional unit of BMI increases the frequency of GI complaints by 11%, and an increase in altitude from 10 to 2,000 m doubles the frequency of these complaints. Concomitant therapy with statins reduces the frequency of GI complaints by about 80%.

The association of anaemia with GI complaints in patients on MTF therapy is not surprising, given that first MTF causes vitamin B_12_ deficiency and consequent macrocytic anaemia [10] and then that hypochromic anaemia is often caused by diseases of the gastroduodenal mucosa (peptic ulcer, gastritis), which make the mucosa more sensitive to the action of exogenous substances reaching a high concentration in the GI secretion after oral intake [11]. In our study, we did not have the insight in the laboratory results of the patients, so we could not determine whether the anaemia that the patients had was macrocytic (caused by vitamin B12) or hypochromic, microcytic (caused by bleeding from the lining of the GI tract), and confirm previous assumptions. Also, other authors have so far not found a connection between anaemia and GI complaints due to MTF use, which indicates that additional studies are necessary to confirm and explain this connection.

After GI infections, a number of patients experience chronic inflammation of the GI tract, with various complaints, which sometimes turns into post-infection irritable bowel syndrome [12]. In such a situation, the application of any drug that can further worsen the functioning of the GI tract will be accompanied by a higher frequency of complaints in that region, which is most likely to happen with the use of MTF. It is known that MTF leads to the accumulation of lactate in the mucosa of the GI tract because it gives priority to the anaerobic metabolism of glucose in the mucosa due to the very high concentration it achieves in the tissue [6]. A high level of lactate creates acidosis locally in the mucous membrane, which stimulates the contraction of smooth muscles and creates a sensation of pain. All these changes will be more pronounced and have more unfavourable consequences when the mucous membrane is already damaged by previous GI infections.

A study on patients from China [13] found no influence of BMI equal to or greater than 25 kg/m^2^ on the frequency of GI complaints in patients on MTF therapy. In our study as well, univariate analysis did not associate BMI with the occurrence of GI complaints, but after adjusting for the effects of other factors in multivariate analysis, an increase in BMI significantly increased the likelihood of GI complaints. The difference in the obtained effects is most likely due to the higher statistical power of the study when BMI is taken as a continuous rather than a categorical variable (greater or less than 25 kg/m^2^). People who are overweight or obese have more frequent GI symptoms, primarily due to unhealthy habits when eating, like aerophagia, swallowing unchewed food, fast eating, and eating or drinking large volumes of food. They frequently complain of bloating, abdominal pain, retching, vomiting, diarrhoea, or incomplete evacuation, which are symptoms often encountered in patients taking MTF, too [14].

Since both drugs, MTF and statins, affect glucose metabolism as well as lipid metabolism, it is not surprising that MTF–statin combination therapy is prescribed to many patients with type 2 diabetes mellitus. In recent years, several studies have been conducted that indicate the positive effects of combined therapy with MTF and statins on various diseases, such as cardiovascular diseases, some cancers, as well as in the treatment of polycystic ovaries [15,16]. There is a study that confirms the finding that the simultaneous use of statins and MTF shows a positive effect on GI side effects. A higher percentage of MTF-tolerant patients used statins (66%) compared to MTF-intolerant patients (48%) [7]. The answer is probably related to the ability of statins to affect the gut microbiota by directly affecting the number of gut bacteria and bile acid metabolism in the gut [17]. Research conducted on mice also shows a positive effect of one statin (rosuvastatin) on the composition and diversity of intestinal microbiota, bile acid metabolism, and immunity of the GI tract [18].

GI problems at high altitude are commonplace [19]. The impact can be explained through the influence of hypoxia at higher altitude on the physiological changes in the digestive system, which can further result in altered absorption, distribution, metabolism, and excretion of drugs. The increase in GI complaints with the use of MTF in people living at higher altitudes can be explained by the effect of hypoxia on slowing down the metabolism of MTF, by increasing both the mean retention time and the half-life time (*t*1/2) of MTF. A study conducted in rats after exposure to simulated hypoxia at high altitude revealed significant changes in the pharmacokinetics of MTF. The key effect of hypoxia is reflected in the reduction of the expression of organic cation transporter 2, which leads to a significant increase in the *t*1/2 of MTF [20].

A possible mechanism by which MTF causes GI AE probably includes stimulation of adenosin-monophosphate-activated protein kinase and consequent inhibition of the mammalian target of rapamycin (mTOR). The mTOR-regulated pathway is responsible for protein synthesis and cell proliferation in normal circumstances; therefore, MTF induces apoptosis of cells in GI epithelium [21–24]. The effect of MTF in our study could have been augmented by pharmacokinetic interactions with drugs that inhibit its membrane transporters OCT1, MATE1, and MATE2K [25]. Although an increase in oxidative stress may have a certain role in GI AEs of MTF, too [26], roles of prohibitin 1 and β-catenin cannot be excluded [27,28]. Since antioxidants phenylethanoid glycoside verbascoside and beta-carotene effectively protect renal podocytes and subcellular structures involved in glucose metabolism from free radicals, we may speculate that the use of antioxidants in general could ameliorate GI AEs of MTF, at least in some patients [29,30].

If one considers the possible relationship between the independent predictors of GI AEs of MTF, hypoxia could be the common denominator. While increased altitude and anaemia both directly contribute to tissue hypoxia, obesity (i.e., increased BMI) is associated with obstructive sleep apnea and consequent chronic intermittent hypoxia [31]. Hypoxia causes chronic activation of hypoxia-inducible factors in the GI tract, which lead to tissue injury and inflammation, making intestines more sensitive to additional stimuli like MTF [32]; a similar causal relationship exists between intestinal infection and MTF.

Our study has several limitations. First of all, due to the attachment of the researchers to certain community pharmacies, the sample could not be random, which opens up the possibility of bias in the selection of respondents. Second, the data collected by the survey could not have been verified in the patients’ medical records. Also, the relatively limited number of subjects made it impossible to detect more subtle influences of potential predictors, which, taken together, can change the overall picture of the conditioning of GI complaints during MTF therapy.

In conclusion, each case of MTF intolerance should be carefully investigated for risk factors, since some of them could be neutralized and the patient prevented from being derived from this very useful drug. Anaemia could be corrected, reinstitution of MTF could be attempted later after a GI infection, the drug could be introduced more gradually in patients living at high altitudes, and sometimes introduction of a statin for some other reason may be helpful.
